# Genome‐wide profiling of circulating tumor DNA depicts landscape of copy number alterations in pancreatic cancer with liver metastasis

**DOI:** 10.1002/1878-0261.12757

**Published:** 2020-07-15

**Authors:** Tao Wei, Jian Zhang, Jin Li, Qi Chen, Xiao Zhi, Wei Tao, Jingjiao Ma, Jiaqi Yang, Yu Lou, Tao Ma, Xiang Li, Qi Zhang, Wei Chen, Risheng Que, Shunliang Gao, Xueli Bai, Tingbo Liang

**Affiliations:** ^1^ Department of Hepatobiliary and Pancreatic Surgery School of Medicine the First Affiliated Hospital of Zhejiang University Hangzhou China; ^2^ Zhejiang Provincial Key Laboratory of Pancreatic Disease Hangzhou China; ^3^ Innovation Center for the study of Pancreatic Diseases Hangzhou China; ^4^ The Scientific and Technical Department Novogene Bioinformatics Institute Beijing China

**Keywords:** circulating tumor DNA, copy number alterations, liver metastasis, pancreatic ductal adenocarcinoma, whole‐genome sequencing

## Abstract

Cell‐free DNA (cfDNA) offers an alternative to tissue biopsies for genomic profiling in tumors. Here, we sought to evaluate copy number alterations in PDAC through whole‐genome sequencing (WGS) of cfDNA and determine their clinical significance. Using shallow WGS across 90 plasma samples from 70 pancreatic cancer patients, we detected somatic copy number alterations (CNAs) in 34 subjects (48.6%). Additionally, a higher tumor fraction (TFx) was associated with increased carbohydrate antigen 19‐9 (CA19‐9), metastasis, and a worse prognosis. Serial cfDNA analysis suggested that CNAs were highly concordant even for progressive disease after chemotherapy. TFx dynamics were largely in line with changed CA19‐9 levels and tumor burden following chemotherapy. Notably, patients with more abundant, baseline CNAs exhibited a better response to chemotherapy. In conclusion, shallow WGS for cfDNA enables a high‐throughput characterization of CNAs and an estimation of tumor burden in metastatic pancreatic cancer. These findings reinforce our understanding of the genomic evolution of metastatic PDAC and might have clinical relevance for guiding treatment.

## Graphical abstract

We used whole‐genome sequencing of cell‐free DNA (cfDNA) to obtain copy number alteration (CNA) profiles from the plasma of patients with metastatic pancreatic cancer (PC). These profiles were compared with TCGA‐derived CNA profiles of primary pancreatic tumors. CNA analysis enabled the prediction of tumor burden and tumor prognosis, and assessment of therapeutic response and clonal evolution.

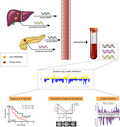

**Abbreviations**
CA19‐9: carbohydrate antigen 19‐9
cfDNA: cell‐free DNA
CNAs: copy number alterations
CR: complete response
ctDNA: circulating tumor DNA
MAF: mutant allele fraction
PD: progressive disease
PDAC: pancreatic ductal adenocarcinoma
PR: partial response
RECIST: Response Evaluation Criteria in Solid Tumors
SD: stable disease
TCGA: The Cancer Genome Atlas
TFx: tumor fraction
WGS: whole‐genome sequencing

## 1 Introduction

Pancreatic ductal adenocarcinoma (PDAC) is among the most lethal malignancies globally [1]. It is projected to be the second leading cause of cancer mortality by 2030 in Western countries [2]. Only minimal increase for median survival of PDAC patients was achieved along with clinical and scientific efforts over the past decades [3]. Nonetheless, subgroup of patients with specific molecular signature had remarkable benefit to treatment like DNA damage‐based therapeutics for BRCA‐mutated patients [4, 5]. This highlights the importance of molecular characterization and personalized management to further refine clinical decision‐making [6, 7].

Pancreatic ductal adenocarcinoma harbors extensive genomic aberrations and complex mutational landscape. Detailed genomic analysis identified a number of recurrently mutated genes that aggregate into ten distinct molecular pathways [8]. Another study revealed four subtypes of PDAC based on structural variations of chromosomes conferring therapeutic relevance [4]. Besides, several transcriptomic profiling work revealed overlapped cancer subtypes that have prognostic impact and are potentially predictive of therapy response [7, 9, 10]. Yet our knowledge on clinical correlation of pancreatic cancer subtyping remains limited. The inaccessibility of tumor specimens with adequate cellularity further constitutes a major impediment in implementation of genomics‐driven precision medicine, in particular for metastatic lesions.

Circulating cell‐free DNA (cfDNA) refers to degraded nucleic acids shed from cells into blood. The cfDNA released by malignant cells is called circulating tumor DNA (ctDNA) and offers an alternative option for tissue biopsy in interrogating genetic alterations [11, 12]. As ctDNA is believed to randomly derive from necrotic and apoptotic tumor cells across the whole tissue, it thus presumably provides a more representative genomic picture of tumor than regional tissue‐based strategies. Numerous studies have shown that ctDNA profiling was able to identify and track cancer‐specific mutations, which is helpful in diagnosis, prediction of prognosis, and treatment response, and guiding personalized therapeutics [11, 13, 14, 15]. Emerging evidence suggested the ability of genome‐wide or exome‐wide ctDNA sequencing to capture genetic diversity including aberrations of copy number and chromosome structure in addition to single nucleotide variants [16, 17].

Somatic copy number alterations (CNAs) are frequently observed in cancer and affect a large fraction of genome [18]. Many protein‐coding genes and noncoding RNAs that located in amplified or deleted regions are cancer drivers and have phenotypic consequences. A number of studies have found that CNAs involving oncogenes and tumor suppressor genes are key events during carcinogenesis, metastatic spreading, and tumor evolution [19]. Here, we interrogate the copy number landscape through low‐coverage whole‐genome sequencing (WGS) of ctDNA in a metastatic PDAC cohort and determine clinical impact of inferred tumor fractions (TFx) and CNAs. Characterization of CNAs in this metastatic setting is also compared with those of primary PDAC tumor tissues derived from the publicly available datasets The Cancer Genome Atlas (TCGA).

## 2 Materials and Methods

### 2.1 Patients

Blood samples were collected between January 2017 and April 2019 from patients who were diagnosed as PDAC at the Second Affiliated Hospital of Zhejiang University, School of Medicine, and the First Affiliated Hospital of Zhejiang University, School of Medicine. Patients with unresectable PDAC of either being locally advanced or having liver metastasis were included. Patients were eligible for this study if at least 1 mL plasma was available. Informed consent was obtained from all subjects, and the study was conducted under the approval of Institutional Review Board. Diagnosis of PDAC was confirmed by pathological examination of either tumor biopsies or surgically removed tissues. Relevant demographics, clinicopathological, and treatment data were collected prospectively. Follow‐up was performed every four and six cycles of chemotherapy (2 months) for patients receiving FOLFIRINOX and gemcitabine plus nab‐paclitaxel regimen, respectively. Assessment of treatment response was done via abdominal enhanced computed tomography and magnetic resonance imaging. According to the Response Evaluation Criteria in Solid Tumors (RECIST) criteria, measurement of tumor response by imaging was quantitatively classified into four categories: complete response (CR), partial response (PR), stable disease (SD), and progressive disease (PD). This work was conducted in accordance with the guideline of the Declaration of Helsinki.

### 2.2 Blood sample processing and cfDNA preparation

Peripheral venous blood samples (5 mL) were harvested in EDTA collections tubes. Blood samples were processed within 4 h of collection. Briefly, plasma and peripheral mononuclear cells were separated by standard density gradient centrifugation of 3000***g*** for 20 min at room temperature. Further centrifugation of 12 000***g*** for 5 min at 4 °C was performed to deplete cell debris within plasma. Isolated plasma was aliquoted and stored in −80 °C. For cfDNA extraction, 1 mL frozen plasma samples were thawed at room temperature. cfDNA were extracted using MagMAX Cell‐free DNA Isolation Kit (ThermoFisher Scientific, Waltham, MA, USA) according to the manufacturer’s instructions. The quantity of extracted cfDNA was measured by using Qubit 2.0 Fluorometer. The size distribution of cfDNA length was determined via Agilent Bioanalyzer 2100, and samples with enrichment at 160 bp indicate typical characteristics of cfDNA.

### 2.3 Whole‐genome sequencing for cfDNA

A total amount of 20 ng cfDNA for each sample was used as input material for library preparations. Sequencing library was generated using Truseq Nano DNA HT Sample Prep Kit (Illumina USA) following the manufacturer’s recommendations, and index codes were added to each sample. Briefly, DNA were end‐polished, A‐tailed, and ligated with the full‐length adapter for Illumina sequencing, followed by further polymerase chain reaction (PCR) amplification. After PCR products were purified (AMPure XP system), libraries were analyzed for size distribution and quantified by real‐time PCR. The libraries were sequenced on Illumina Hiseq platform. An average genome‐wide fold coverage of 5 × was expected to be obtained. Valid sequencing data were mapped to the reference human genome hg19 by Burrows‐Wheeler Aligner (BWA) software. All the downstream bioinformatical analyses were based on the high‐quality clean data, which had removed reads containing adapter, reads containing Ploy‐N, and low‐quality reads from raw data.

### 2.4 Somatic CNAs calling

Copy number analysis and TFx estimation were performed using ichorCNA method as previously described [20]. Briefly, the genome was divided into T nonoverlapping windows, or bins, of 1 Mb. Aligned reads were counted based on overlap within each bin using the tools in HMMcopy Suite (http://compbio.bccrc.ca/software/hmmcopy/). The read counts were then normalized to correct for GC content and mappability biases, and then, CNAs and TFx were estimated. Genes were defined as gain (corresponds to three copies), amplification (corresponds to four or more copies), or deletion (corresponds to one copy) versus diploid (corresponds to two copies).

### 2.5 TCGA data processing

TCGA data for primary pancreatic cancer tissues (*n* = 184) were downloaded using firehose provided by the Broad Institute. Somatic CNA data were derived from Affymetrix SNP arrays (Affymetrix Genome‐wide Human 6.0), and precomputed segmented log2 ratio was used for the analyses. For comparison of copy number profile, only tissues with high tumor cellularity were selected as previously reported [21].

### 2.6 Gene‐level copy number analyses

GISTIC2.0 output was used for all gene‐level copy number analyses. Segmented data files derived from ichorCNA analysis for cfDNA and TCGA data were purity and ploidy corrected, then input into GISTIC2.0 with amplification/deletion threshold log2 ratio > 0.3, confidence level 0.99, and *Q*‐value threshold 0.25.

### 2.7 Statistical analysis

Mann–Whitney *U*‐test and chi‐squared test were used for comparison of continuous parameters and categorical variables, respectively. Correlation analysis was performed using Pearson correlation method. Survival analysis was performed using Kaplan–Meier method. All statistical calculations were performed using spss 22.0 (SPSS, Chicago, IL, USA) and prism 6.0 (GraphPad, La Jolla, CA, USA). Two‐sized *p* value less than 0.05 was considered as significant.

## 3 Results

### 3.1 Metastatic PDAC cohort

**Fig. 1:**
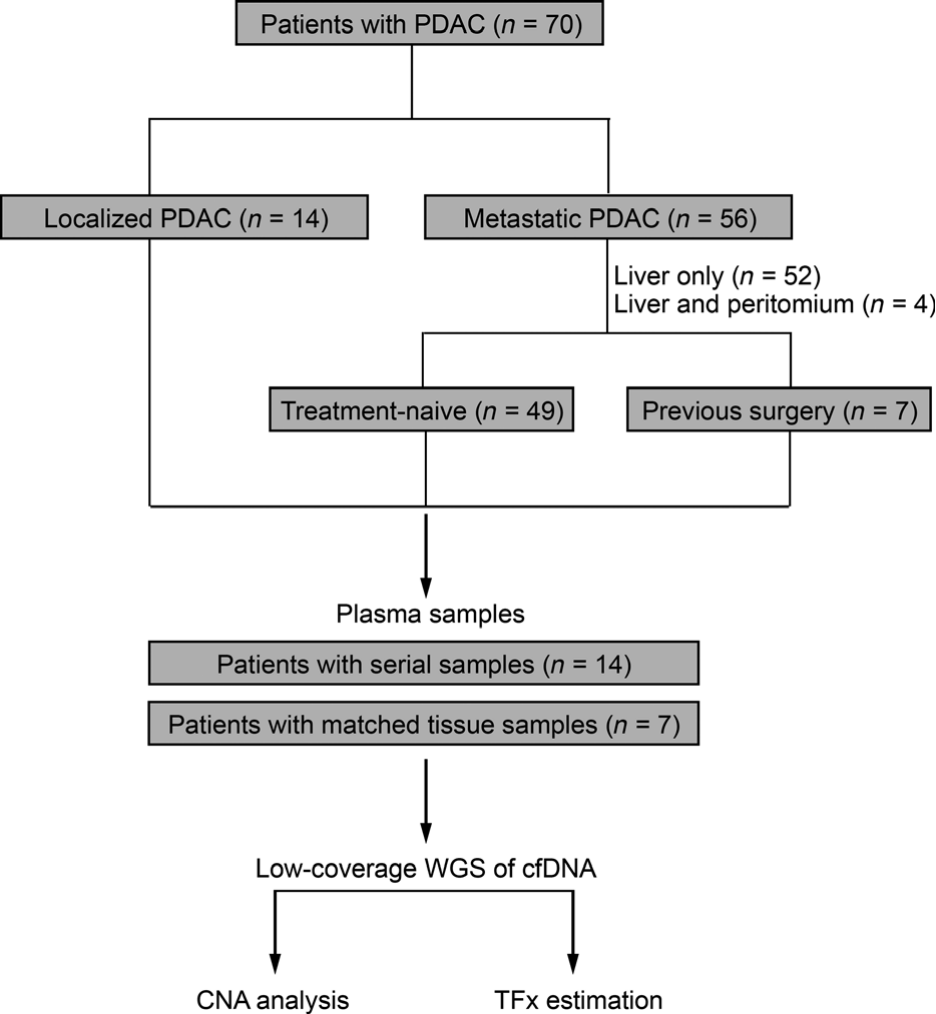
Schema of the clinical cohort of pancreatic cancer. PDAC, pancreatic ductal adenocarcinoma; WGS, whole‐genome sequencing; cfDNA, cell‐free DNA; CNAs, copy number alterations; TFx, tumor fractions.

Ninety plasma samples in total harvested from 70 patients with pathology‐proven PDAC were processed for low‐coverage WGS (Fig. 1). Fourteen patients had either one (*n* = 8) or two (*n* = 6) serial plasma samples available following chemotherapy. The majority of cases were treatment‐naïve at the time of first blood collection (63/70; 90%), including 49 patients with metastasis and 14 with localized disease. Other seven subjects had received surgical resection and adjuvant chemotherapy. For these patients, baseline blood samples were collected when liver metastasis was found postoperatively, and the median duration from surgery to development of metastasis was 8 months (range, 1 to 21 months). Longitudinal serial plasma samples were procured for 14 individuals during treatment course. For metastatic patients, the median time of follow‐up from diagnosis of metastasis was 12 months (range, 1 to 20 months).

### 3.2 Shallow whole‐genome sequencing of cfDNA enables tumor fractions estimation

**Fig. 2:**
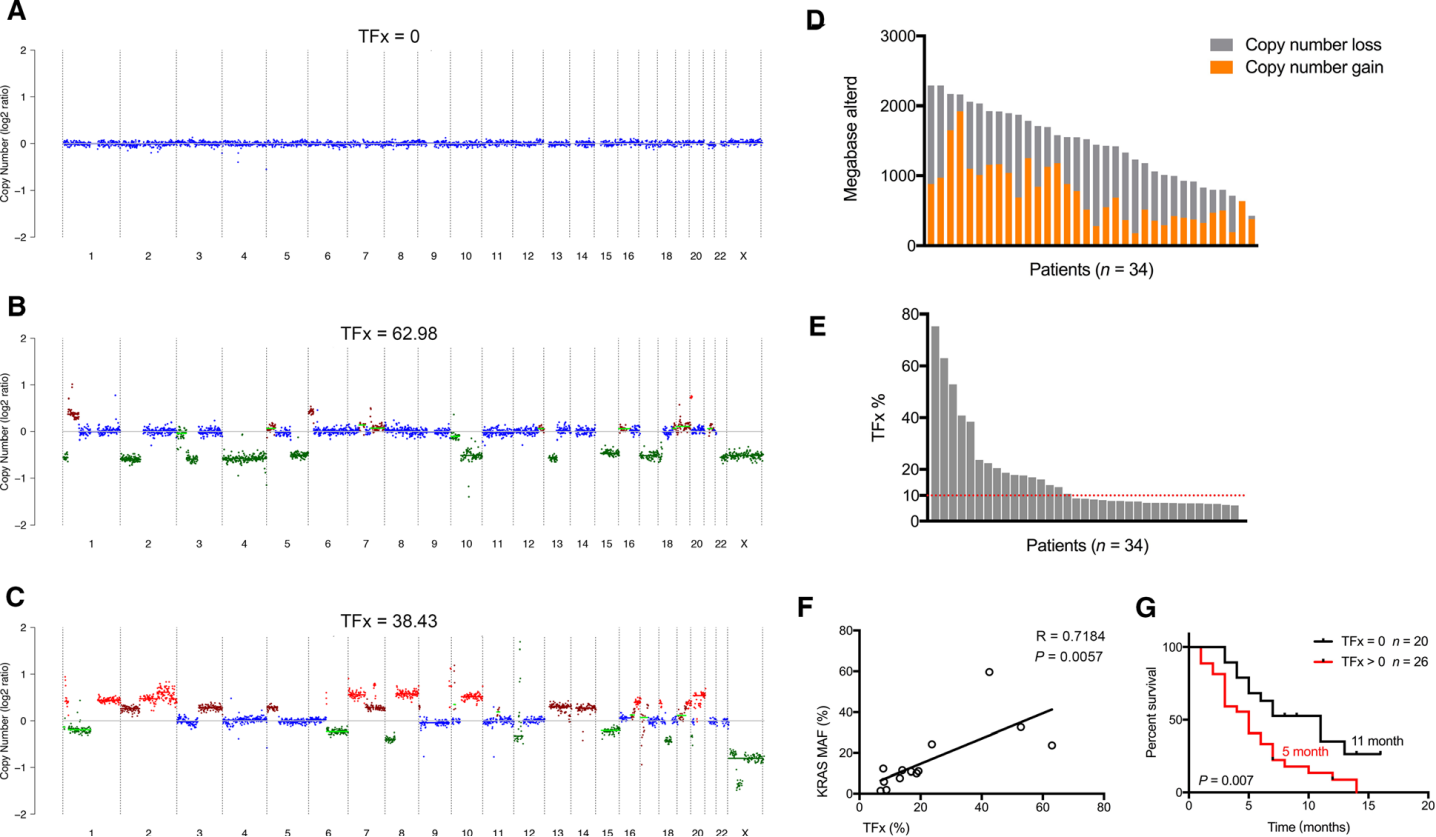
Shallow whole‐genome sequencing of cfDNA enables characterization of somatic CNAs and estimation of TFx. (A–C) Examples of copy number landscape inferred by cfDNA analysis. (D) Size of copy number gain and loss for each case with detectable CNAs at baseline. (E) Distribution of TFx estimated from cfDNA profiling for patients with detectable CNAs at baseline. (F) Correlation analysis of TFx and mutant allele fractions for altered *KRAS*. (G) Survival comparison between patients with TFx = 0 and TFx> 0.

Our low‐coverage WGS generated a median sequencing depth of 5.2× (range, 4.2–5.8×) for cfDNA from all subjects at baseline. Overall, somatic CNAs were observed in thirty‐four subjects (48.6%, 34/70). Extensive aberrations were found across chromosomes focally, segmentally, and numerically with remarkable heterogeneity between individuals (Fig. 2A–D). On average, 23.8% (~738 Mb) and 22.8% (~707 Mb) of the genome exhibited copy number gain and loss for these cases, respectively (Fig. 2D).

Shallow WGS of cfDNA enables estimation of TFx through ichorCNA analysis. For patients with detected CNAs in our cohort, TFx was calculated as ranging from 6.1% to 75.3% (Fig. 2E), whereas those without detectable alterations had TFx of 0. *KRAS* alteration had been previously determined by targeted genomic sequencing for a subset of patients in the current cohort. The mutant allele fractions of *KRAS* were found to be well correlated with TFx (Fig. 2F). As sequencing depth might affect data interpretation and previous studies mostly adopted ultra‐low‐pass sequencing of 0.1×, we analyzed the copy number signature of patients by comparing several distinct depths, that is, 0.1×, 0.25×, 0.5×, 1×, 3×, and 5×. The results suggested nearly identical TFx for each sample when analyzed by above distinct depths (Table S1). However, higher sequencing depth generated a better resolution of gene‐level copy number landscape (Fig. S1).

Then, we sought to evaluate the clinicopathological determinants of CNAs detection or TFx > 0. Release of tumor‐derived DNA into circulation is linked to tumor burden. As expected, all 30 patients with detection of CNAs had liver metastasis in contrast localized disease where no CNAs were found. Moreover, patients with TFx over 0 exhibited markedly higher serum level of conventional tumor markers like carbohydrate antigen 19‐9 (CA19‐9) level compared with those with TFx of 0 (Table 1). Demographic factors, histology, and radiographic characteristics including lymph node involvement and vascular invasion were not linked to increased TFx (Table 1). Higher TFx was associated with shortened overall survival (Fig. 2G) and was the only statistically significant prognostic factor for prognosis (Table S2).

**Table 1:** Correlation between TFx and clinical characteristics. LN, lymph node; TFx, tumor fractions

Variables	All patients (*n* = 63)	TFx		*P* value
		0 % (*n* = 33)	>0 % (*n* = 30)	
Demographics				
Age, median (range)	64 (43–86)	66 (44–78)	63 (43–86)	0.538
Gender, n				
Male	41	21	20	0.801
Female	22	12	10	
Smoking status, n				
No	39	19	20	0.458
Yes	24	14	10	
**Radiographic features**				
Tumor location, n				
Proximal	22	13	9	0.435
Distal	41	20	21	
Liver metastasis, n				
No	12	12	0	**<0.001**
Yes	51	21	30	
LN enlargement, n				
No	15	11	4	0.063
Yes	48	22	26	
Arterial invasion, n				
No	16	10	6	0.311
Yes	44	21	23	
Vein invasion, n				
No	13	9	4	0.152
Yes	47	22	25	
Tumor markers				
CA19‐9, median (range)	1908.4 (2–12 000)	448	7582	**0.001**
CEA, median (range)	6.8 (0.6–846.8)	3.4	11.9	**0.015**
CA12‐5, median (range)	85.8 (10.7–3871.9)	53.0	24	**0.024**
Pathological information				
Biopsy location, n				0.106
Primary tumor	34	21	13	
Liver metastasis	29	12	17	
Differentiation, n				
Well and moderate	25	12	13	0.279
Poor	26	13	13	
Adenosquamous	6	5	1	

The bold values represent p value less than 0.05.

### 3.3 CNA landscape of metastatic PDAC inferred by cfDNA analysis

**Fig. 3:**
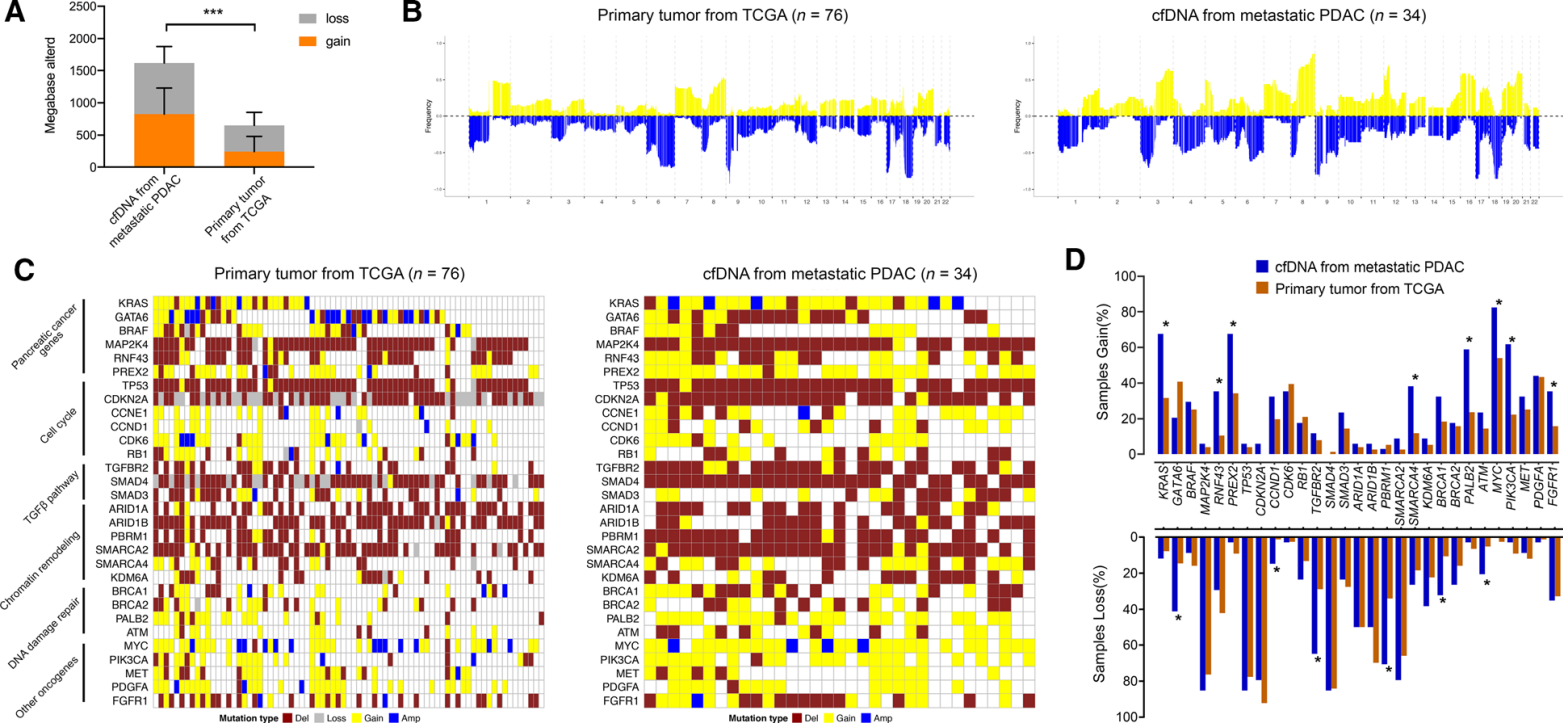
Depiction of CNAs landscape in the metastatic and primary settings of PDAC. (A) Total size of CNAs for primaries in TCGA (copy number gain: 242 ± 239 bp, copy number loss: 412 ± 197 bp) and cfDNA derived from metastatic PDAC (copy number gain: 823 ± 409 bp, copy number loss: 793 ± 260 bp). Two‐way ANOVA, ^***^ *P* < 0.001; the error bar represents standard deviation (SD). (B–D) The frequency of gene‐level alterations and per‐sample CNAs for 30 pancreatic cancer‐related genes in primary and metastatic setting. ^*^Fisher’s exact false discovery rate adjusted *P* < 0.05

To determine the patterns of chromosomal alterations of metastatic PDAC, cfDNA samples with TFx > 10% or containing well‐defined CNAs were selected for high‐confidence copy number calls (*n* = 34). On the other hand, we further included patients who had primary tumor tissues profiled in TCGA database to compare copy number characteristics of primary PDAC with metastatic PDAC. Only tissues with high tumor cellularity were included for CNAs identification (*n* = 76). We found that metastatic PDAC contained significantly increased percent of genome with both copy number gain and loss in contrast to primaries (Fig. 3A). Overall, the altered genomic regions were largely concordant between two groups in both chromosomal and gene level, but most regions demonstrated higher frequency of CNAs in metastatic group than primary samples (Fig. 3B, Figure S2). At the level of individual genes, a subset of PDAC‐related genes exhibited significantly greater frequency of alterations in metastatic patients including gain of *KRAS* and *MYC* and deletion of *TGFBR2* and *PBRM1* among others (Fig. 3C,D).

### 3.4 Assessment of CNAs in plasma and matched tumor tissues

**Fig. 4:**
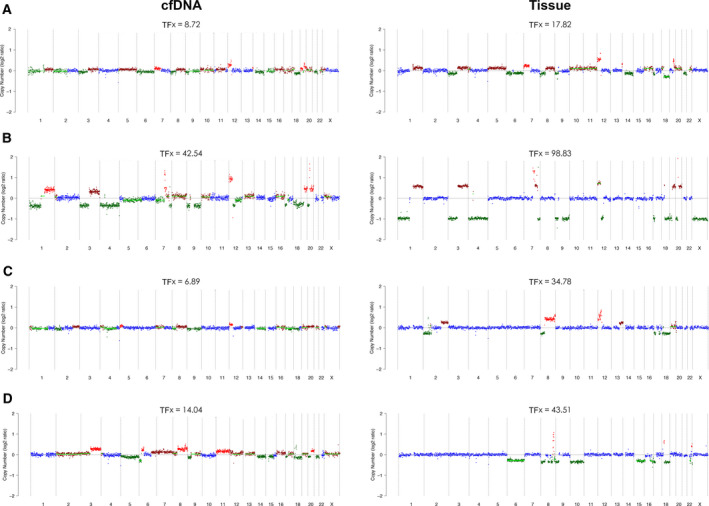
Analysis of CNAs in cfDNA and matched tumor tissues for four individuals (A–D)

CNAs derived from plasma samples and tumor tissues were compared in six individuals. In the first case, synchronous liver metastatic tissue and plasma were obtained at the same time, and copy number landscape is nearly identical albeit a low TFx for cfDNA (Fig. 4A). Other five patients underwent surgical resection and liver metastasis developed postoperatively with a median time interval of 5 months (range, 2–8), for whom CNAs of primary tumor tissues and postoperative cfDNA were compared. Among them, TFx estimated from ichorCNA analysis was zero for two cases due to low tumor cellularity. For the remaining three pairs, we observed a strong concordance of profiles between tissue and plasma in two subjects (Fig. 4B). But there were increased CNAs in cfDNA in contrast to tissue particularly subclonal alterations. In the last case, CNA landscape of cfDNA was remarkably divergent with much more broad regions altered compared with tissue‐derived DNA. These data suggested the possibility of genomic evolution with respect to CNAs during metastatic progression of pancreatic cancer.

### 3.5 Serial cfDNA profiling and cancer genome evolution

To explore genomic evolution and potential resistance mechanisms upon chemotherapy, longitudinal serial ctDNA analysis was performed in 14 patients. All patients completed at least one course therapy of modified FOLFIRINOX or gemcitabine plus nab‐paclitaxel regimen, and treatment response was evaluated by cross‐sectional imaging. Dynamic changes of TFx derived from cfDNA profiling were in line with clinical response for 13 subjects. In contrast, for the last patient, TFx reduced in spite of increase of tumor burden after chemotherapy (Table 2). Interestingly, we found that genome‐wide copy number profiles were not significantly altered upon chemotherapy exposures using the above sample pairs (Fig. S3). A few inconsistent CNAs for each individual were mainly subclonal alterations which were possibly missed for samples with low TFx. Of note, we found that the high abundance of CNAs is associated with favorable response to chemotherapy, whereas the TFx did not correlate with therapy response (Fig. S4A,B).

**Table 2:** Dynamics of TFx following chemotherapy. Nab‐P: Nab‐paclitaxel; Gem: gemcitabine; PD: progressive disease; SD: stable disease; PR: partial response

Patient No.	Chemotherapy regimen	TFx (%)		CA19‐9 (U·mL^−1^)		Therapy response
		Before therapy	After therapy	Before therapy	After therapy	
1	Nab‐P plus Gem	0	13.07	671.2	1414.9	PD
2	mFOLFIRINOX	52.82	8.56	12000	2676.7	PR
3	mFOLFIRINOX	0	13.53	75.3	52.3	PD
4	Nab‐P plus Gem followed by mFOLFIRINOX	13.23	0	11.5	20.5	SD
5	Nab‐P plus Gem followed by mFOLFIRINOX	8.36	15.3	11115.3	12000	PD
6	Nab‐P plus Gem	14.04	6.15	12000	12000	PD
7	Nab‐P plus Gem	16.94	39.43	2016.1	486.3	PD
8	mFOLFIRINOX	6.89	10.5	7364.8	4916.7	PD
9	mFOLFIRINOX	0	0	4783.7	5357.5	SD
10	Nab‐P plus Gem followed by mFOLFIRINOX	7.81	19.19	2	2	PD
11	mFOLFIRINOX	6.68	13.82	12000	12000	PD
12	mFOLFIRINOX	0	5.98	12000	2541.3	PD
13	Nab‐P plus Gem followed by mFOLFIRINOX	7.09	19.34	898.3	10906.2	PD
14	mFOLFIRINOX	0	0	3.6	21.2	SD

**Fig. 5:**
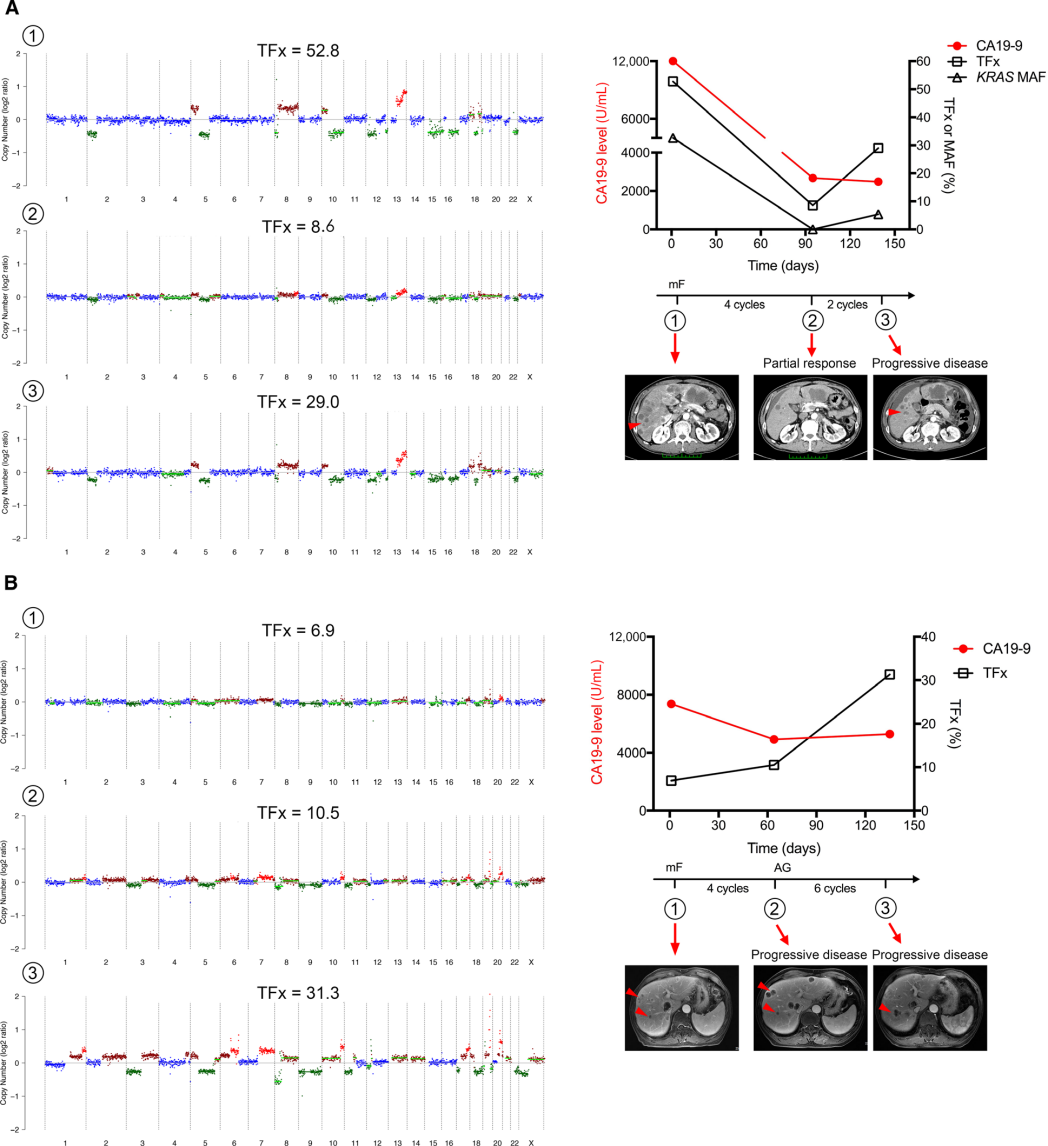
Longitudinal serial cfDNA profiling following chemotherapy in two patients (A,B). The left panel shows the CNA pattern in each time point following chemotherapy corresponding to the time points in the right panel. The right panel shows the dynamics of CA19‐9 level, TFx, MAF for *KRAS*, and tumor burden on imaging in each time point following chemotherapy. The arrowheads indicate metastatic lesions in liver. MAF: mutant allele fraction, mF: modified FOLFIRINOX, AG: Nab‐paclitaxel and gemcitabine.

We further highlighted two cases with multiple plasma samples analyzed. The first patient exhibited favorable response to FOLFIRINOX initially but progression developed after 6 cycles of treatment. This clinical context was consistent with dynamics of TFx and mutant allele fraction for *KRAS* (Fig. 5A). In the second case, continuous progression was observed following either FOLFIRINOX or gemcitabine plus nab‐paclitaxel treatment. The dynamics of TFx was in line with clinical findings with sustained elevation (Fig. 5B). Characteristics of CNAs were nearly identical through the treatment course for these two patients (Fig. 5A,B).

## 4 Discussion

Circulating cfDNA holds great promise as a serum biomarker for clinical applications. Numerous studies have reported cfDNA analysis in pancreatic cancer by targeted sequencing of genomic regions of interest to profile mutations of oncogenes [22]. Here, we investigated the copy number landscape of pancreatic cancer via whole‐genome sequencing of cfDNA. The present whole‐genome analysis enables identification of characteristic CNAs and estimation of TFx for PDAC in the metastatic setting with clinical implications.

Genomic alterations of pancreatic cancer have been extensively profiled by sequencing of primary tumors [21]. Yet mutational landscape for metastasis remains unclear because metastatic tissues are not routinely removed or biopsied. Several studies reported collection of concomitant primary and metastatic tissues in a small number of PDAC patients through rapid autopsy. Exome‐ and genome‐wide sequencing of paired samples suggested largely concordant alterations particularly driver gene mutation [23, 24]. A recent study included nearly 300 PDAC patients with paired or unpaired primaries and metastasis and confirmed the conserved genomic signatures including point mutation, CNAs, and structural alterations [25]. Our cfDNA‐exclusive analysis of liver metastasis also showed a similar CNA patterns compared with those of primary tissues derived from TCGA database. Notably, cfDNA‐based genomic characterization theoretically provides a more representative full spectrum of alterations compared with tissue sequencing, which is in particular important in the context of multiple metastatic lesions. These data are in agreement with previous findings of ovarian carcinoma that genome‐wide copy number change mainly reflects historic evolutions acquired during tumorigenesis [26]. But our study and others also showed that metastasis owns distinct genomic features like increased frequency of altered copy number in a few chromosomal regions like amplification of 12p12.1 covering *KRAS* [25]. This indicated that CNAs evolution might contribute to tumor metastatic progression. Interestingly, we found completely divergent copy number profiles in one patient with matched primary and metastatic samples. A few other studies demonstrated genomic heterogeneity between primary and metastatic tumors and that progression of metastasis required genetic alterations beyond those essential for primaries [27]. This implies that genomic evolutions including CNAs are critical at least for some patients during metastasis development.

Until now, the only confirmed predictor of chemotherapy efficacy is mutations of DNA damage repair genes BRCA1 and BRCA2 to platinum‐based therapies [6, 28]. We demonstrated that patients with broader region of CNAs had more favorable response to chemotherapy. This correlation requires further validation of large cohort and understanding of underlying mechanisms. Consistently, one recent study indicated that chromosomal instability assessed through copy number analysis of ctDNA correlated with chemotherapy response in gastric cancer [29]. Other studies also showed that high copy number load predicts favorable outcome of targeted therapy or immunotherapy through whole‐genome sequencing of either tumor tissues or cell‐free DNA [30, 31]. In addition, it is reported that CNAs could evolve under treatment pressure and contribute to acquired therapy resistance [32, 33]. For example, copy number gain of androgen receptor is critical for progression of prostate cancer and is associated with castration resistance. Serial cfDNA analysis revealed more prominent amplification level of androgen receptor following castration therapy [33]. In the current cohort, CNA patterns are not significantly changed after chemotherapy even for patients with progressive disease. This indicated a largely stable copy number profile and is in line with the notions that CNAs are predominantly occurred at early stage of tumor development. Nevertheless, it is possible that de novo focal gains or losses give rise to resistant subclones.

Shallow genome‐wide sequencing provides a way for estimation of tumor burden [20]. Several studies adopted this approach in breast cancer, lung cancer, prostate carcinoma, and neuroblastoma and found the estimated tumor fractions are clinically relevant [33, 34, 35, 36, 37, 38]. We found that TFx was remarkably higher in the metastatic setting than localized tumor. This discrepancy of TFx might be simply due to increased tumor burden for advanced‐stage disease. On the other hand, the release of nucleic acids into bloodstream is linked to tumor growth rate [39]. Two studies have shown that metastatic tissues were more proliferative than primary counterparts [25, 40], which may be responsible for the increased TFx. In addition, our data validated the prognostic significance of estimated TFx as found in other cancers. Of note, TFx of pancreatic cancer in our study is lower than other types of cancer. This is consistent with a recent report that pancreatic cancer is among the low ctDNA cancers [41]. Emerging evidence indicated that tumor‐derived cfDNA were shorter than those from nonmalignant cells, and enrichment of short fragments could improve detection of ctDNA [41, 42]. Indeed, we also found that estimated TFx for samples with selection of short reads after sequencing was significantly higher than that of original samples (data not shown). This highlights proper optimization of the genome‐wide sequencing approach in the future analysis of cfDNA.

The present study has a few limitations. First, our sample size was small and the characterization of genomic landscape might be insufficient given a fraction of samples with low TFx. Second, the presence of primary tumor might compromise the interpretation of genomic landscape of metastasis when performing cfDNA profiling. Further studies by recruiting patients with solely liver metastasis should reconcile this problem and provide a clearer picture of genomic change. Third, genetic heterogeneity was found between distinct metastatic lesions especially for different organs, and phylogenetic trees showed organ‐specific branches. Our study predominantly included patients with liver metastasis, and whether unique CNAs features are present for metastasis to other sites like lung and peritoneum requires future delineation.

## 5 Conclusions

In summary, the present study revealed genome‐wide CNA pattern through cfDNA profiling in liver metastatic PDAC. This strategy allows identification of copy number change and tumor fractions and owns the potential to advance our understanding of metastasis, chemotherapy resistance, and novel therapeutic approach.

## Author contributions

TL, TW, and XB conceived and designed the data. TW, JZ, JL, QC, XZ, XL, TM, WC, SG, RQ, XB, and TL acquired the data. TW, WT, JM, JY, YLu, QZ, and TL analyzed and interpreted the data. TWi, JZ, and TL wrote the manuscript. All authors reviewed the manuscript.

## Conflicts of Interest

The authors declared no conflict of interest.

## Supporting information


**Fig S1.** Example of copy number profile analyzed by different sequencing depth.

**Fig S2.** GISTIC analysis for copy number profiles of cfDNA derived from metastatic PDAC (A) and primary tumor tissues in TCGA database (B).

**Fig S3.** Comparison of CNAs in patients before and after chemotherapy. The upper plot and lower plot refer to pre‐treatment and post‐treatment in each panel, respectively.

**Fig S4.** The correlation between chemotherapy response and CNAs load or TFx. NS: not significant; PD: progressive disease; PR: partial response; SD: stable disease.

**Table S1.** Comparison of TFx using different sequencing depth.

**Table S2.** Analysis of potential risk factors of overall survival in metastatic PDAC.

Supplementary Material

## Data Availability

Raw sequence data have been deposited at the Sequence Read Archive (SRA) under accession number PRJNA633741.
