# Supplementary figures and images for: Genome‐wide profiling of circulating tumor DNA depicts landscape of copy number alterations in pancreatic cancer with liver metastasis
Source: Mol Oncol. 2020 Jul 15;14(9):1966–77. doi: 10.1002/1878-0261.12757 (PMC7463305; doi:10.1002/1878-0261.12757)

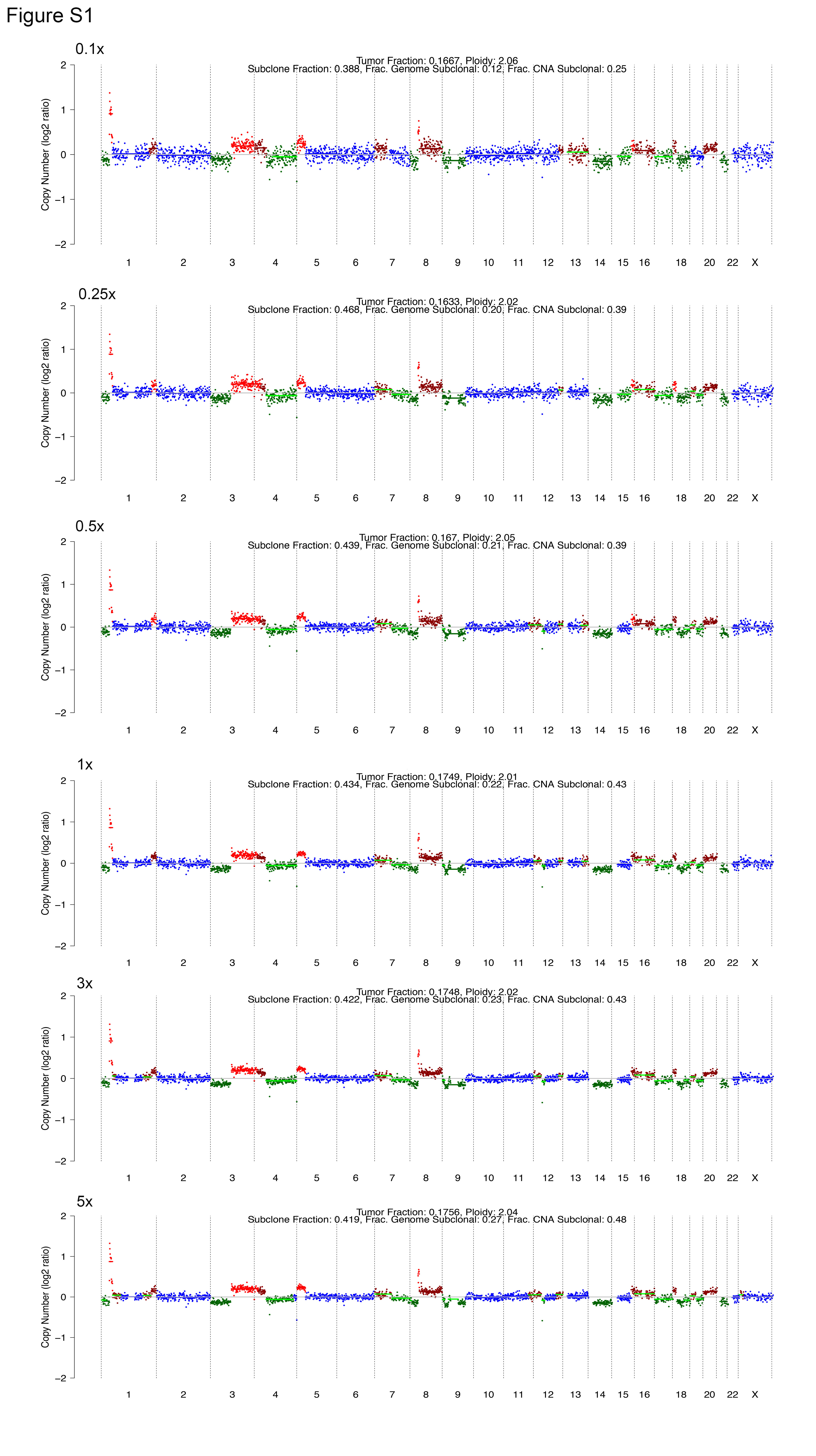

Supplement: Supplementary file 1 — Fig S1. Example of copy number profile analyzed by different sequencing depth. [file MOL2-14-1966-s001.jpg]

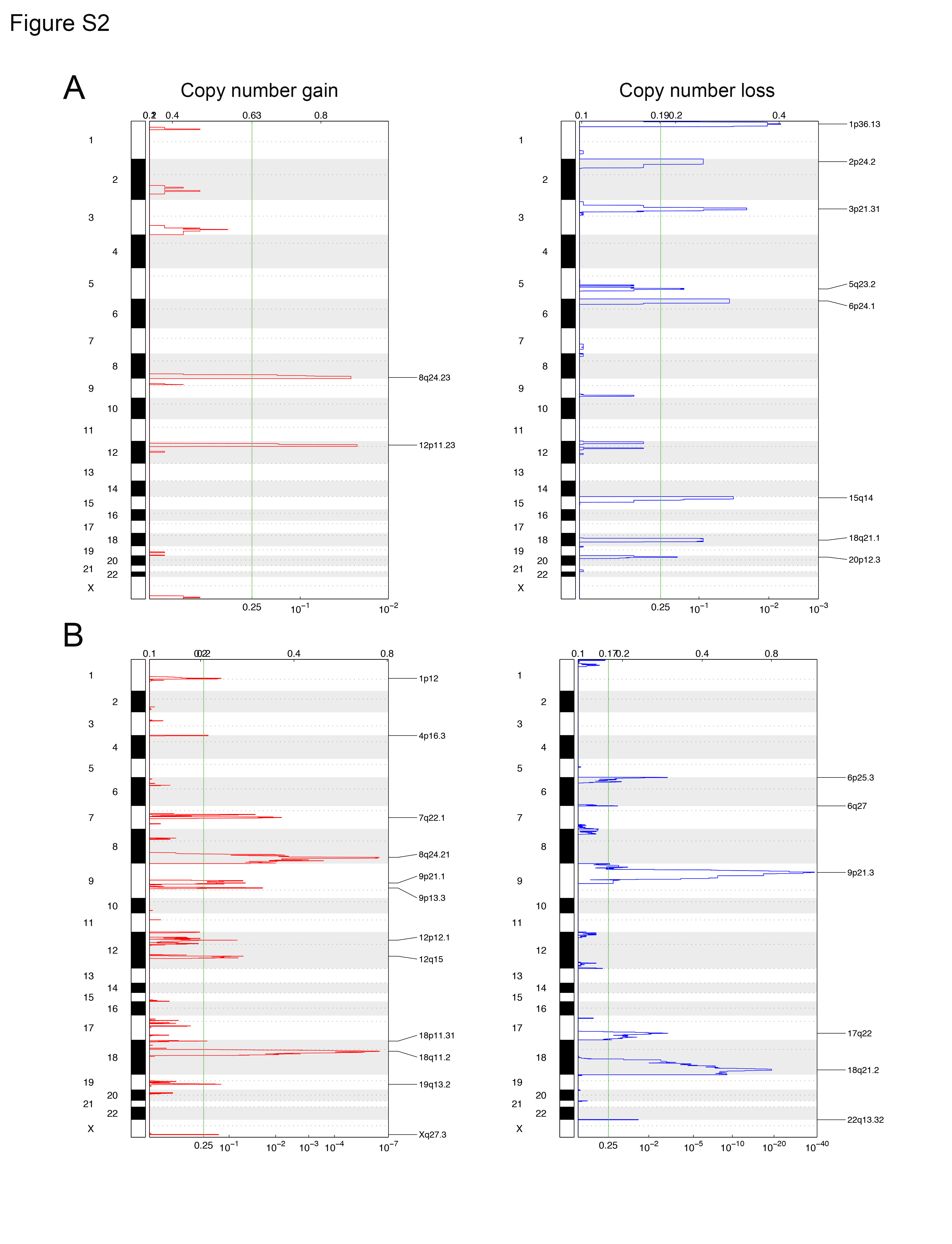

Supplement: Supplementary file 2 — Fig S2. GISTIC analysis for copy number profiles of cfDNA derived from metastatic PDAC (A) and primary tumor tissues in TCGA database (B). [file MOL2-14-1966-s002.jpg]

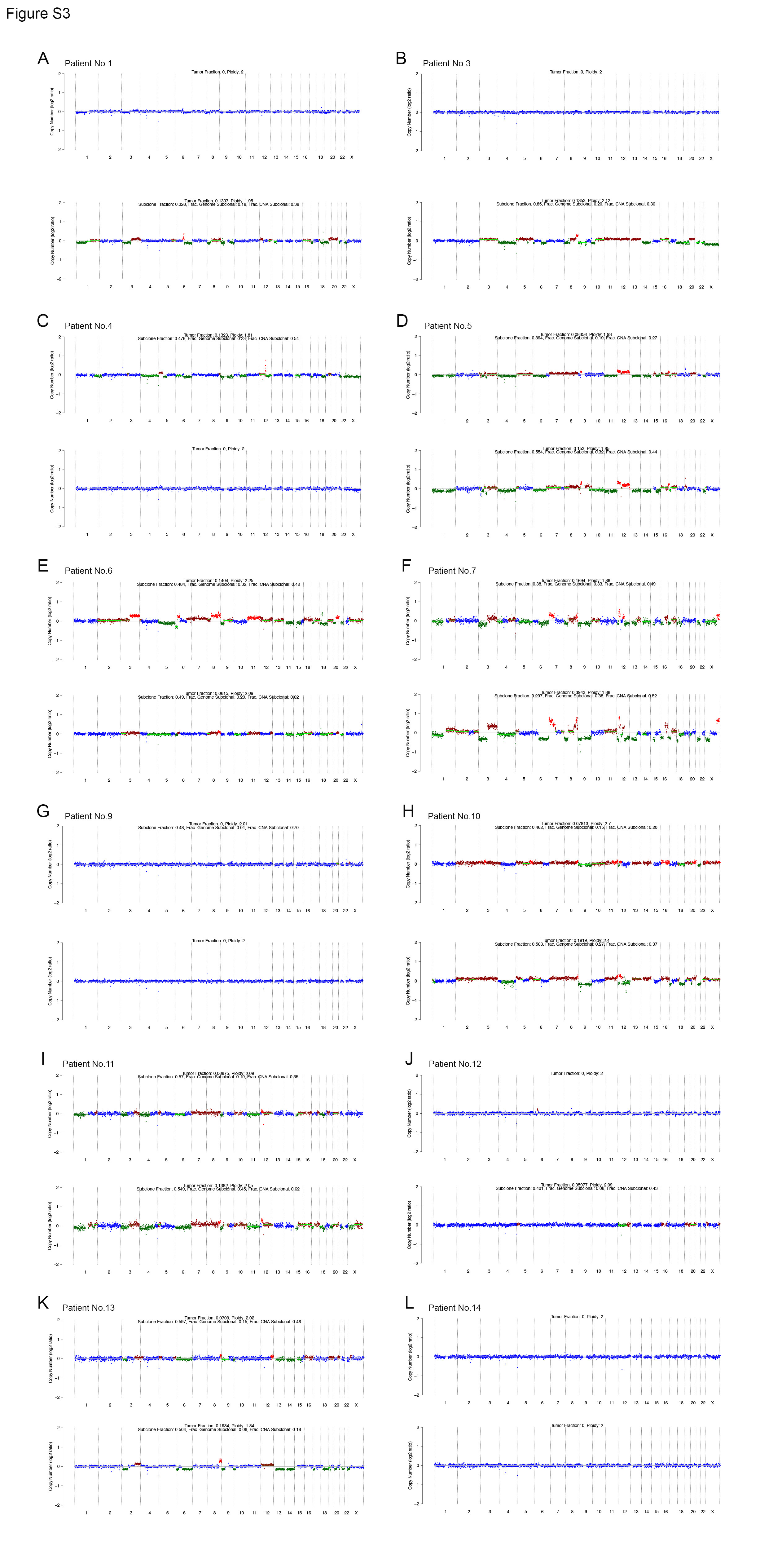

Supplement: Supplementary file 3 — Fig S3. Comparison of CNAs in patients before and after chemotherapy. The upper plot and lower plot refer to pre‐treatment and post‐treatment in each panel, respectively. [file MOL2-14-1966-s003.jpg]

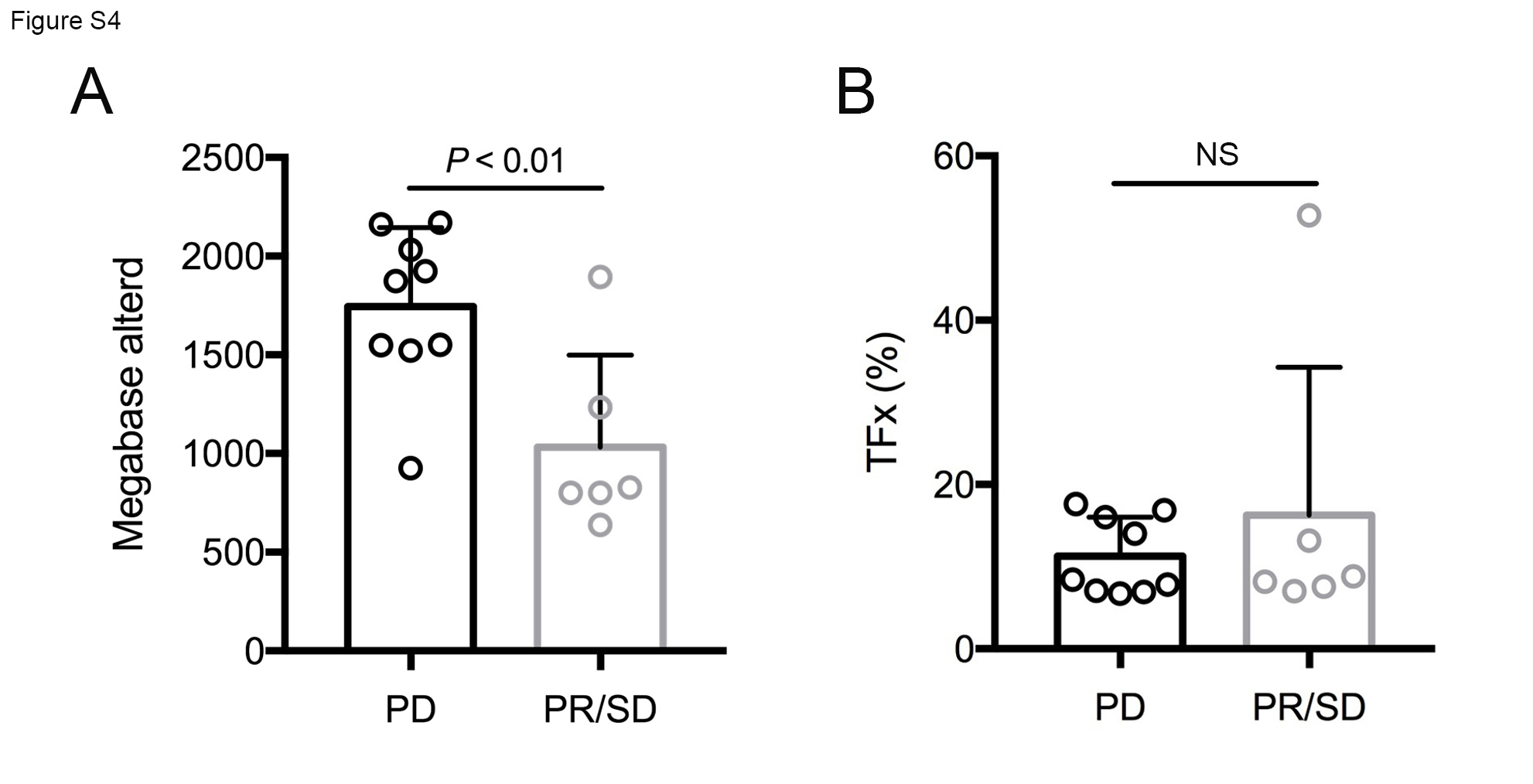

Supplement: Supplementary file 4 — Fig S4. The correlation between chemotherapy response and CNAs load or TFx. NS: not significant; PD: progressive disease; PR: partial response; SD: stable disease. [file MOL2-14-1966-s004.jpg]
