# Supplementary material for: Genome‐wide profiling of circulating tumor DNA depicts landscape of copy number alterations in pancreatic cancer with liver metastasis
Source: Mol Oncol. 2020 Jul 15;14(9):1966–77. doi: 10.1002/1878-0261.12757 (PMC7463305; doi:10.1002/1878-0261.12757)
Supplement: Supplementary file 5 — Table S1. Comparison of TFx using different sequencing depth. [file MOL2-14-1966-s005.docx]

Supplementary table 1. Comparison of TFx using different sequencing depth.

| Case | TFx | | | | | |
| --- | --- | --- | --- | --- | --- | --- |
|  | 0.1× | 0.25× | 0.5× | 1× | 3× | 5× |
| 1 | 40.75 | 40.25 | 40.38 | 40.54 | 40.73 | 40.82 |
| 2 | 14.65 | 13.15 | 13.24 | 13.4 | 13.06 | 13.07 |
| 3 | 20.42 | 14.11 | 17.58 | 13.82 | 14.0 | 14.04 |
| 4 | 16.67 | 16.33 | 16.7 | 17.49 | 17.48 | 17.56 |
| 5 | 11.72 | 8.3 | 7.86 | 7.63 | 7.57 | 7.61 |
| 6 | 14.95 | 10.28 | 10.29 | 14.58 | 14.38 | 10.5 |
| 7 | 32.62 | 39.48 | 39.39 | 39.39 | 33.3 | 39.43 |
| 8 | 10.14 | 14.98 | 14.45 | 15.24 | 15.24 | 15.3 |
| 9 | 18.99 | 19.11 | 19.24 | 19.36 | 19.31 | 19.34 |
| 10 | 30.47 | 30.78 | 30.49 | 30.9 | 31.5 | 31.28 |
