# Supplementary material for: Genome‐wide profiling of circulating tumor DNA depicts landscape of copy number alterations in pancreatic cancer with liver metastasis
Source: Mol Oncol. 2020 Jul 15;14(9):1966–77. doi: 10.1002/1878-0261.12757 (PMC7463305; doi:10.1002/1878-0261.12757)
Supplement: Supplementary file 6 — Table S2. Analysis of potential risk factors of overall survival in metastatic PDAC. [file MOL2-14-1966-s006.docx]

Supplementary Table 2. Analysis of potential risk factors of overall survival in metastatic PDAC.

| Variable | *P* value |
| --- | --- |
| Age, <65 vs >65 | 0.490 |
| Gender, Male vs Female | 0.929 |
| Smoking, Yes vs No | 0.366 |
| Tumor location, Proximal vs Distal | 0.578 |
| Lymph node involvement, Yes vs No | 0.839 |
| Arterial invasion, Yes vs No | 0.445 |
| Vein invasion, Yes vs No | 0.178 |
| CA19-9, >2000 vs <2000 | 0.232 |
| TFx, >0 vs =0 | 0.007 |
