# Supplementary material for: Genome‐wide profiling of circulating tumor DNA depicts landscape of copy number alterations in pancreatic cancer with liver metastasis
Source: Mol Oncol. 2020 Jul 15;14(9):1966–77. doi: 10.1002/1878-0261.12757 (PMC7463305; doi:10.1002/1878-0261.12757)
Supplement: Supplementary file 7 — Supplementary Material [file MOL2-14-1966-s007.docx]

**Supporting Information**

**Supplementary Figure S1.** Example of copy number profile analyzed by different sequencing depth.
**Supplementary Figure S2.** GISTIC analysis for copy number profiles of cfDNA derived from metastatic PDAC (A) and primary tumor tissues in TCGA database (B).

**Supplementary Figure S3.** Comparison of CNAs in patients before and after chemotherapy. The upper plot and lower plot refer to pre-treatment and post-treatment in each panel, respectively.

**Supplementary Figure S4.** The correlation between chemotherapy response and CNAs load or TFx. NS: not significant; PD: progressive disease; PR: partial response; SD: stable disease.

**Supplementary Table S1.** Comparison of TFx using different sequencing depth.

**Supplementary Table S2.** Analysis of potential risk factors of overall survival in metastatic PDAC.
